# Impact of Illness on Electronic Health Use (The Seventh Tromsø Study - Part 2): Population-Based Questionnaire Study

**DOI:** 10.2196/13116

**Published:** 2020-03-05

**Authors:** Luis Marco-Ruiz, Rolf Wynn, Sunday Oluwafemi Oyeyemi, Andrius Budrionis, Kassaye Yitbarek Yigzaw, Johan Gustav Bellika

**Affiliations:** 1 Norwegian Centre for E-health Research University Hospital of North Norway Tromsø Norway; 2 Department of Clinical Medicine Faculty of Health Sciences UiT The Arctic University of Norway Tromsø Norway; 3 Division of Mental Health and Addictions University Hospital of North Norway Tromsø Norway; 4 Department of Community Medicine Faculty of Health Sciences UiT The Arctic University of Norway Tromsø Norway

**Keywords:** eHealth, internet, search engines, mobile apps, social media

## Abstract

**Background:**

Patients who suffer from different diseases may use different electronic health (eHealth) resources. Thus, those who plan eHealth interventions should take into account which eHealth resources are used most frequently by patients that suffer from different diseases.

**Objective:**

The aim of this study was to understand the associations between different groups of chronic diseases and the use of different eHealth resources.

**Methods:**

Data from the seventh survey of the Tromsø Study (Tromsø 7) were analyzed to determine how different diseases influence the use of different eHealth resources. Specifically, the eHealth resources considered were use of apps, search engines, video services, and social media. The analysis contained data from 21,083 participants in the age group older than 40 years. A total of 15,585 (15,585/21,083; 73.92%) participants reported to have suffered some disease, 10,604 (10,604/21,083; 50.29%) participants reported to have used some kind of eHealth resource in the last year, and 7854 (7854/21,083; 37.25%) participants reported to have used some kind of eHealth resource in the last year and suffered (or had suffered) from some kind of specified disease. Logistic regression was used to determine which diseases significantly predicted the use of each eHealth resource.

**Results:**

The use of apps was increased among those individuals that (had) suffered from psychological problems (odds ratio [OR] 1.39, 95% CI 1.23-1.56) and cardiovascular diseases (OR 1.12, 95% CI 1.01-1.24) and those part-time workers that (had) suffered from any of the diseases classified as others (OR 2.08, 95% CI 1.35-3.32). The use of search engines for accessing health information increased among individuals who suffered from psychological problems (OR 1.39, 95% CI 1.25-1.55), cancer (OR 1.26, 95% CI 1.11-1.44), or any of the diseases classified as other diseases (OR 1.27, 95% CI 1.13-1.42). Regarding video services, their use for accessing health information was more likely when the participant was a man (OR 1.31, 95% CI 1.13-1.53), (had) suffered from psychological problems (OR 1.70, 95% CI 1.43-2.01), or (had) suffered from other diseases (OR 1.43, 95% CI 1.20-1.71). The factors associated with an increase in the use of social media for accessing health information were as follows: (had) suffered from psychological problems (OR 1.65, 95% CI 1.42-1.91), working part time (OR 1.35, 95% CI 0.62-2.63), receiving disability benefits (OR 1.42, 95% CI 1.14-1.76), having received an upper secondary school education (OR 1.20, 95% CI 1.03-1.38), being a man with a high household income (OR 1.67, 95% CI 1.07-2.60), suffering from cardiovascular diseases and having a high household income (OR 3.39, 95% CI 1.62-8.16), and suffering from respiratory diseases while being retired (OR 1.95, 95% CI 1.28-2.97).

**Conclusions:**

Our findings show that different diseases are currently associated with the use of different eHealth resources. This knowledge is useful for those who plan eHealth interventions as they can take into account which type of eHealth resource may be used for gaining the attention of the different user groups.

## Introduction

### Background

This is the second paper of a series of 4 that studies electronic Health (eHealth) consumption using the data gathered by the seventh survey of the Tromsø Study (Tromsø 7). In the study by Wynn et al (part 1) [1], we present main findings regarding characteristics of the participants and their use of eHealth. In this second paper, we focus on understanding how long-term or chronic diseases influence the choice of one eHealth resource over another. In the study by Budrionis et al (part 3) [2], we examine outcomes of the use of eHealth, and in the study by Yigzaw et al (part 4) [3], we study how eHealth consumption influences actual doctor visits.

The overall aim of the series was to provide a clearer overview of the characteristics of eHealth users and their interaction with the health care sector. As a matter of fact, the health care sector in many developed economies is facing challenges that include aging populations, lack of workforce, and insufficient coordination among caregivers and services [4-7]. At the same time, the use of information and communication technology is increasing among citizens [8-10]. In the United States, 84% of the population has access to the internet [8]. In Norway, 85% of the population uses the internet on a daily basis [11]. The increase in the use of technology is also powered by the broad access to mobile phones and tablets. In 2012, 85% of US adults owned a mobile phone and 31% had used it to look for health information [12]. The broad adoption of smartphones and ubiquitous access to the internet have led to a steady increase in the use of technology that may be used for health purposes, such as search engines, social media, and Web-based video services [13-17].

eHealth can be understood as the “intersection of medical informatics, public health and business, referring to health services and information delivered or enhanced through the internet and related technologies” [18]. Access to technology allows citizens to easily access health information and monitor their health status with, for example, mobile apps. It is known that chronic conditions can influence the use of the internet for seeking health information [9,19-22]. The appropriate use of technology has the potential to improve patients’ health and make them more knowledgeable about their condition [23-25]. However, the vast amount of health-related information available on the internet also includes irrelevant information and misinformation [26-28]. Typical challenges for patients with chronic disorders when looking for health information on the internet are finding appropriate online resources and filtering online health information [29].

### Interventions and Challenges of Electronic Health

Currently, many health trusts are promoting eHealth interventions [30-34]. These interventions focus on eHealth resources such as mobile apps, social media, video services, and search engines on the internet, among others [8-10]. These interventions have focused on improving health care by guiding health consumers to the most appropriate service [35-37], improving treatment adherence [30,31], or involving patients in shared decision making [38]. A strong focus on these interventions has been set on long-term and chronic diseases such as cardiovascular diseases, cancer, and psychological problems [31,33,39].

Some studies have shown that technology can improve treatment adherence for chronic patients [30,31]. Examples are the positive impact of mobile apps and social media on the management of chronic diseases such as diabetes and epilepsy [30,40]. Most studies have focused on studying the effect of eHealth resources on the management of a condition. However, another important aspect that has been less explored is how a particular condition predisposes to the use of one type of eHealth resource over another. This knowledge is important to decide what type of eHealth resource is the most appropriate for every eHealth intervention. However, to our knowledge, the importance of diseases for eHealth use has not been explored with a sufficient sample size to find which eHealth resources are preferred by different groups of chronic patients.

Tromsø 7 included a questionnaire about the use of eHealth. In a series of 4 papers, we explore data from the Tromsø Study questionnaire analyzing the relationships among eHealth use and other demographic and clinical variables. The large sample size of the Tromsø 7 offers the opportunity to compare eHealth preferences in different patient groups.

## Methods

### The Tromsø Study

The Tromsø Study is a longitudinal population-based study conducted in the municipality of Tromsø, Norway, since 1974 [41,42]. Its original purpose was to determine the reasons for the high mortality due to cardiovascular diseases in Norway. However, over time, it has expanded, and currently, it covers many different diseases such as mental disorders, cancer, and osteoporosis, to name a few [42]. The study is funded directly by the Norwegian Government. The study is conducted by the University of Tromsø in collaboration with the Norwegian Institute of Public Health and others [42]. The most recent version is Tromsø 7 , comprising the years 2015 and 2016 [42]. The Tromsø Study focuses on a range of chronic diseases and conditions. In Tromsø 7, people aged 40 years or older were included, which provided a sample of 21,083 participants accounting for 64.69% (21,083/32,591) of the total invited. A personal invitation was mailed to all residents in Tromsø aged 40 years or older [41,42] together with a paper-based questionnaire and a link to an electronic questionnaire. Those who chose to participate could complete the questionnaire in paper or electronically at home. Alternatively, they could do so when they attended the study center, where they were also included in other tests. Those who did not respond to the initial mailed invitation were mailed a follow-up reminder.

Part 1 of this series of papers has already presented the characteristics of the participants in Tromsø 7 [1].

### Questionnaire

The questionnaire in Tromsø 7 included data regarding many diseases, symptoms, and lifestyle and contained in total more than 300 questions. Examples of the data included are dietary habits; medication; sleeping patterns; socioeconomic status; education; work; and, the most relevant for this study, the use of eHealth resources.

The eHealth questions were selected based on a review of prior literature and with a particular focus on prior studies involving Norwegian participants. As there were strict limits on the number of items (because of the overall size of the questionnaire), only the main questions regarding eHealth services were included (as described below).

The Tromsø 7 questionnaire completed by participants contained several blocks of information. In this study, we focused on a subset of the information contained in the questionnaire. The information considered in this study is as follows:

- Demographics: including questions about age, gender, education, household income (expressed in Norwegian kroner [kr] and US dollars), lifestyle, and occupation.
- Groups of diseases: the participant suffers or has suffered from a cardiovascular disease (high blood pressure, heart attack, heart failure, atrial fibrillation, angina, and stroke), respiratory disease (bronchitis and asthma), cancer, psychological problems, or other disorders (rheumatoid arthritis, arthrosis, diabetes, kidney disease, migraine, and chronic pain). Participants could choose any of the specific diseases available in the questionnaire (ie, more than 1 disease if relevant). We grouped specific diseases before the statistical analyses were performed.
- Emotional: live with a spouse and support from friends.
- Use of eHealth resources: participants were asked: “How often during the last year have you used the following internet-services for information and advice on health and disease issues: Applications (‘Apps’) for smart phone or tablet?, Search engines (like Google)?, Social media (like Facebook)?, Video services (like YouTube)?” For each question, the participants could answer either “never,” “once,” “a few times,” or “often.” The participants who answered that they had used minimum 1 of the eHealth services were thereafter asked: “If you during the last year have used internet-services for information and advice on health and disease issues, based on the information you found on the internet: Have you decided to go to the doctor?, Have you decided not to go the doctor?, Have you discussed the information with a doctor?, Have you changed your medication without consulting a doctor?, Have you been unsure whether the treatment you have received is correct?, Have you decided to seek out complementary or alternative treatment?, Have you made lifestyle changes?, Have you felt anxiety?, Have you felt reassured?, Have you felt more knowledgeable?, Have you felt more confused?” For each of the questions, the participants could answer either “never,” “once,” “a few times,” or “often.” All the questions and response-options have been published on the Tromsø Study website [43].

### Statistical Analysis

We used multivariable logistic regression to determine which variables influenced the use of eHealth resources. We proceeded in 2 steps. First, a general model predicting the use of any type of eHealth resources was estimated using the whole dataset. For this, we defined a binary variable that indicated if the participant had used any of the eHealth resources or none. The use of each type of eHealth resource (mobile apps, search engines, video services, or social media) was analyzed separately by regressing the dependent variable that represented each type of eHealth resource with the independent variables previously presented. For the second step, we used the subset of patients that had some of the diseases under study (independent of whether or not they used some eHealth resource). In this way, we identified the specific variables that most strongly influenced the use of each type of eHealth resource.

Multimedia Appendix 1 shows the diseases and the eHealth resources considered. In addition, it also shows the demographic variables included in models. Age was treated as a continuous variable. Household income and education were treated as ordinal variables that represented increasing degrees of the feature represented. Occupation was represented as a categorical variable. The groups of diseases considered were coded as dichotomous variables that represented the presence (value=1) or absence (value=0) of any of the diseases included in the group. Similarly, the use and nonuse of different dichotomous resources (mobile apps, search engines, Web videos, and social media) were coded as another dichotomous variable (use=1 and nonuse=0). The sex of the participant was also represented by 0=woman and 1=man. We studied the interactions between age, sex, occupation, education, household income, and the diseases included in the study. All the independent variables were included for the estimation of every model.

Observations with missing data were excluded from the analysis when any of the missing variables (dependent or independent) needed for calculating each logistic regression model were missing. The reader should note that this caused a variation in the total sample available for each specific model, but the procedure maximized the amount of data available for the estimation of each model. This is a common practice to increase the robustness of the statistical model (pairwise exclusion) [44]. We adjusted for covariates by including possible confounders and interactions in the logistic regression models [45]. Models were then simplified excluding nonsignificant variables and interactions. Deviance analysis was performed to check that the models were significant in predicting the use of eHealth resources. All analyses were 2-sided, and *P* values were considered statistically significant at a level of <.05.

### Ethics

The Regional Committee for Medical and Health Research Ethics approved the study (REK Nord, reference 2014/940). All participants provided written informed consent.

## Results

### User Statistics

This section presents the results of the statistical analysis performed on Tromsø 7 data to establish which variables influence the use of each type of eHealth resource. First, this section presents the analysis of eHealth resources as a combined variable that represents any type of eHealth resource (apps, search engines, video services, or social media). Second, this section presents the results of analyzing the relationship between different disease groups and the use of specific eHealth resources for those individuals that suffered from at least one disease. Some interactions are available in Multimedia Appendix 2.

Multimedia Appendix 1 shows the data regarding the demographic characteristics of the sample selected. A total of 15,585 (8565 men, 7020 women) out of 21,083 (73.92%) participants (had) suffered from some kind of disease, 10,604 out of 21,083 (50.29%) participants reported to have used some kind of eHealth resource in the last year, and 7854 out of 21,083 (37.25%) participants reported both to have used some kind of eHealth resource in the last year (apps, search engines, Web videos, or social media) and suffered (or had suffered) from some kind of specified disease. By disease group, of the total 21,083 participants, 34.00% (7169/21,083) participants (had) suffered from some cardiac disease, 7.76% (1636/21,083) from cancer, 12.91% (2723/21,083) from psychological problems, 12.99% (2738/21,083) from respiratory diseases, and 52.69% (11,109/21,083) from any of the diseases included in the others group. More details about the demographic characteristics can be seen in part 1 of this series of studies. In addition, Multimedia Appendix 1 displays the use of eHealth resources per patient group.

### Study of the Use of Electronic Health Resources in General

The first model estimated the use of any eHealth resource. The sample size after removing the respondents that had any missing values was 18,578 individuals. We found that various groups of diseases have a significant effect on the use of eHealth resources. Moreover, different diseases are related to the use of different types of eHealth resources.

Attending to the odds ratios (ORs) in Multimedia Appendix 2, it is possible to see that an increment in age (OR 0.94, 95% CI 0.93-0.95), being a man (OR 0.3, 95% CI 0.21-0.44), living with a spouse (OR 0.84, 95% CI 0.76-0.92), receiving support from friends (OR 0.81, 95% CI 0.73-0.90), and having received college education for less than 4 years (OR 0.88, 95% CI 0.82-0.94) were associated with a decrease in the use of eHealth resources in general.

**Figure 1:**
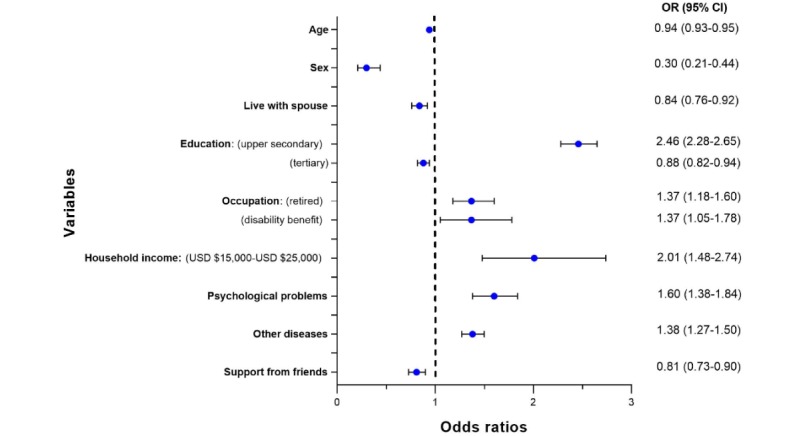
Forest plot for the logistic regression for the use of any electronic health resource.

Figure 1 shows the forest plot summarizing the significant variables that predicted the use of eHealth resources in general (apps, search engines, videos, or social media). The full result of the analysis is available in Multimedia Appendix 2. Having received education of upper secondary school (OR 2.46, 95% CI 2.28-2.65), being retired (OR 1.38, 95% CI 1.18-1.60), receiving a disability benefit (OR 1.37, 95% CI 1.05-1.78), having a household income between US $15,000-$25,000 (OR 2.01, 95% CI 1.48-2.74), suffering from psychological problems (OR 1.60, 95% CI 1.38-1.84), and suffering from any of the diseases contained in the group named *other diseases* (OR 1.38, 95% CI 1.27-1.50) were associated with an increase in the use of eHealth resources in general.

### Study of the Use of Specific Electronic Health Resources

For studying the effect of each disease on the use of eHealth resources, we selected a subsample containing all the participants that suffered from any of the diseases previously presented (n=15,585). Observations containing missing data were only excluded if any of the variables needed for the regression analysis were missing.

#### Study of the Use of Mobile Apps

**Figure 2:**
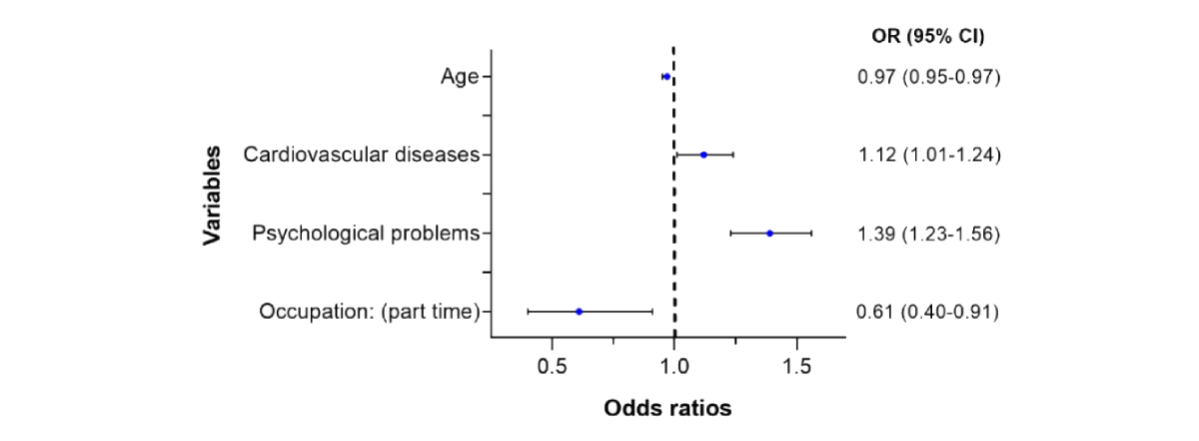
Forest plot for the logistic regression for the use of mobile apps.

The sample size used by the statistical software after removing the observations missing any of the variables used by the mobile apps regression model was 15,321 individuals. Figure 2 summarizes the significant disease groups and demographic characteristics related to the users of apps contained in the subsample. In addition, it contains the ORs from the regression model predicting the use of mobile apps and the influence of each independent variable. The full result of the analysis is available in Multimedia Appendix 3.

A total of 2 main factors were associated with a decrease in the use of mobile apps: age and part-time workers. As age increased, there was a decreasing use of mobile apps (OR 0.97, 95% CI 0.95-0.97). In addition, those included in the work group representing part-time employees were associated with a decrease in the use of mobile apps for accessing health information (OR 0.61, 95% CI 0.40-0.91).

There were 2 main diseases that were associated with an increase in the use of apps for accessing health information: psychological problems (OR 1.39, 95% CI 1.23-1.56) and cardiovascular diseases (OR 1.12, 95% CI 1.01-1.24).

Suffering from any of the diseases contained in the group *other diseases* did not have a significant influence over the use of mobile apps.

#### Study of the Use of Search Engines

**Figure 3:**
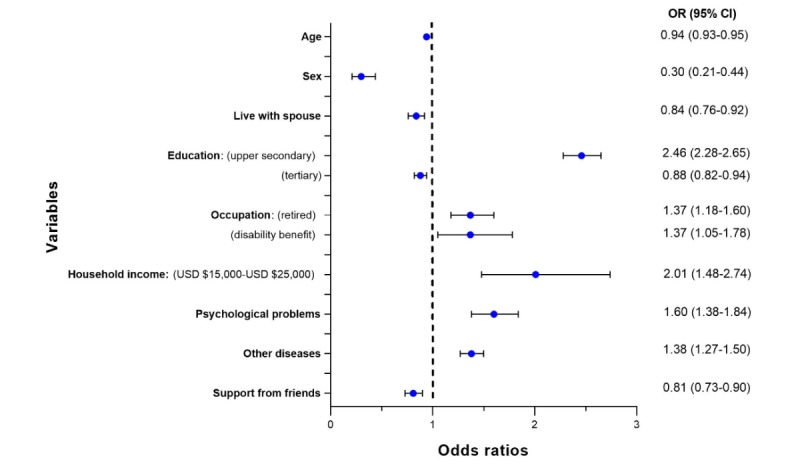
Forest plot for the logistic regression for the use of search engines.

The sample size used by the statistical software after removing the observations missing any of the variables used by the search engines model was 13,610 individuals. Figure 3 summarizes the significant disease groups and demographic characteristics related to the users of search engines contained in the subsample. In addition, it contains the ORs from the regression model predicting the use of Web search engines and the influence of each independent variable. The full result of the analysis is available in Multimedia Appendix 4.

From the logistic regression model, it is possible to interpret that having an educational level of upper secondary education (OR 2.54, 95% CI 2.33-2.77), having a household income of US $15,000-$25,000 (OR 2.57, 95% CI 1.86-3.60), suffering from psychological problems (OR 1.39, 95% CI 1.25-1.55), suffering from cancer (OR 1.26, 95% CI 1.11-1.44), suffering from some of the diseases included in the group *other diseases* (OR 1.27, 95% CI 1.13-1.42), or being retired (OR 1.31, 95% CI 1.07-1.59) contributed to increasing the use of Web search engines for health information. Increasing age (OR 0.94, 95% CI 0.93-0.95), being a man (OR 0.32, 95% CI 0.21-0.50), living with the spouse (OR 0.82, 95% CI 0.73-0.92), having less than 4 years of college education (OR 0.85, 95% CI 0.79-0.92), having support from friends (OR 0.80, 95% CI 0.71-0.90), and having a household income between US $55,100-$75,000 (OR 0.74, 95% CI 0.62-0.87) were associated with a decrease in the use of Web search engines for accessing health information.

#### Study of the Use of Video Services

**Figure 4:**
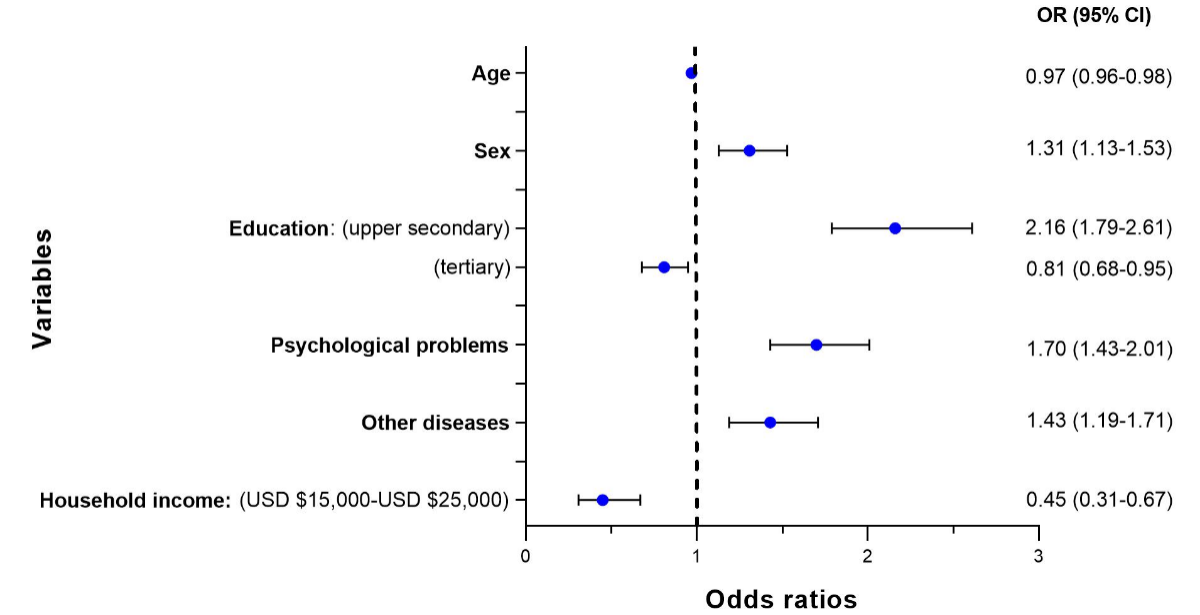
Forest plot for the logistic regression for the use of video services.

The sample size used by the statistical software after removing the observations missing any of the variables used by the model for video services was 14,724 individuals. Figure 4 summarizes the significant disease groups and demographic characteristics related to the users of video services contained in the subsample. In addition, it contains the ORs from the regression model predicting the use of video services and the influence of each independent variable. The full result of the analysis is available in Multimedia Appendix 5.

Having an educational level of upper secondary school (OR 2.16, 95% CI 1.79-2.61), being a man (OR 1.31, 95% CI 1.13-1.53), suffering from psychological problems (OR 1.70, 95% CI 1.43-2.01), and suffering from any of the diseases contained in the group of *others* (OR 1.43, 95% CI 1.19-1.71) were associated with an increase in the use of video services for accessing health information. Increasing age (OR 0.97, 95% CI 0.96-0.98), having an education of less than 4 years of college (OR 0.81, 95% CI 0.68-0.95), and having a household income of US $15,000-$25,000 (OR 0.45, 95% CI 0.31-0.67) were associated with a decrease in the use of video services for accessing health information.

#### Study of the Use of Social Media

The sample size used by the statistical software after removing the observations missing any of the variables used by the model for social media was 14,514 individuals.

**Figure 5:**
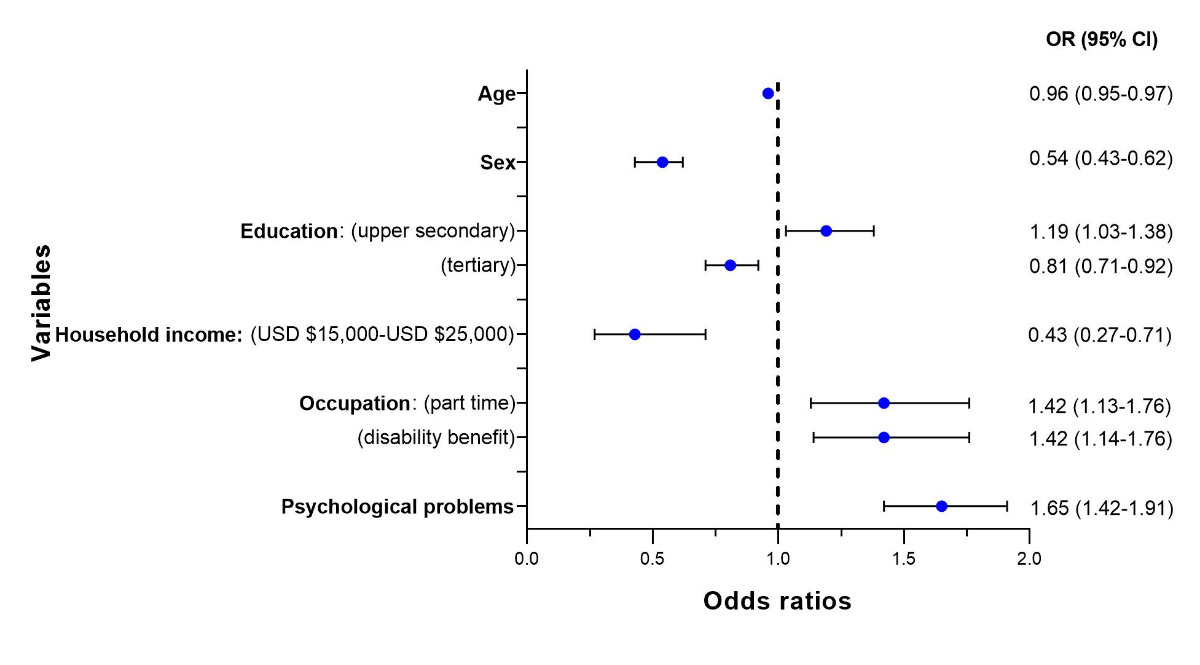
Forest plot for the logistic regression for the use of social media.

Figure 5 summarizes the significant disease groups and demographic characteristics related to the users of social media contained in the subsample. In addition, it contains the ORs from the regression model predicting the use of social media and the influence of each independent variable. The full result of the analysis is available in Multimedia Appendix 6.

Having a part-time job (OR 1.42, 95% CI 1.13-1.76), receiving a disability benefit (OR 1.42, 95% CI 1.14-1.76), having an education level of upper secondary school (OR 1.19, 95% CI 1.03-1.38), and suffering from psychological problems (OR 1.65, 95% CI 1.42-1.91) were associated with an increase in the use of social media for accessing health information.

Higher age (OR 0.96, 95% CI, 0.95-0.97), being a man (OR 0.54, 95% CI, 0.43-0.62), being in the group of those with a household income of US $15,000-$25,000 (OR 0.43, 95% CI 0.27-0.71), and having an education level of less than 4 years of college (OR 0.81, 95% CI 0.71-0.92) were associated with a decrease in the use of social media for accessing health information.

Table 1 shows a summary with the associations that were significant regressing disease groups with eHealth resources, some variables have been omitted for clarity.

**Table 1:** Summary of the association of electronic resources and disease groups.

Disease group	Mobile apps	Search engines	Video services	Social media
Cardiovascular diseases	OR^a^ 1.12, 95% CI 1.01-1.24	—^b^	—	—
Cancer	—	OR 1.26, 95% CI 1.11-1.44	—	—
Psychological problems	OR 1.39, 95% CI 1.23-1.56	OR 1.39, 95% CI 1.25-1.55	OR 1.70, 95% CI 1.43-2.01	OR 1.65, 95% CI 1.42-1.91
Respiratory problems	—	Significant when interacting with a household income of US $55,100 – $75,000^c^	—	Significant when interacting with occupation=retired^c^
Other diseases	Significant when interacting with part-time work^c^	OR 1.27, 95% CI 1.13-1.42	OR 1.43, 95% CI 1.19-1.71	—

^a^OR: odds ratio.

^b^The association between the disease and the electronic resource is not statistically significant.

^c^The interaction is significant. OR for interactions are available in multimedia appendices.

## Discussion

### Summary of Evidence

This study sheds light on the use of various eHealth resources by patients that suffer from diverse conditions. To our knowledge, this is the first study covering the relationship between ranges of different health conditions and varying preferences for different eHealth resources. As depicted in Table 1, our results show that, in general, different diseases are associated with the use of different eHealth resources.

In general, lower socioeconomic class (SES) positively predicted the use of eHealth resources. In addition, for cardiovascular and respiratory diseases, the interaction with lower SES caused an additional increase in the use of social media and search engines, respectively.

Previous studies have shown that people suffering from chronic illness are more likely to search for health information on the internet [9,14]. However, these studies focused on the use of internet for health in general, whereas this study analyzes in depth the use of each type of resource by each group of patients.

Our study shows that long-term and chronic diseases significantly influence the use of eHealth resources. This is consistent with prior research reporting that internet users living with a chronic disease are more likely to gather information using the internet [9,14,19,46]. In addition, this study adds to this knowledge by showing that the influence on each specific eHealth resource varies depending on the medical condition.

Prior literature has shown that most online health information searching starts at a search engine [9]. Furthermore, in our dataset, search engines were the most frequently used eHealth service among those suffering from some disease (7468/15,585; 47.92%), followed by apps (1982/15,585; 12.72%), social media (1145/15,585; 7.35%), and video services (767/15,585; 4.92%). However, in this study, we found that when the use of different eHealth resources is studied independently over the whole dataset, patients with different diseases appear to have variations in preferences regarding different eHealth resources. We believe that these differences in preferences can be partially explained by the availability and popularity of various eHealth resources for the different patient groups—which again might depend in part on characteristics of the different patient groups. For instance, there are many popular apps available for the management of psychological problems [32,39,47,48], such as sleeping problems, anxiety, and depression. In contrast to other chronic disorders, the apps available for psychological problems might even help cure a problem (ie, sleeping problem)—this is obviously not the case, for instance, with cancer or cardiovascular diseases.

Patients with psychological problems were likely to use all the eHealth resources under study (apps, search engines, videos, and social media). Previous studies have reported psychological variables as predictors of health-related internet use [49]. Internet videos have been reported to benefit patients with mental illness [47]. Moreover, internet- and apps-based interventions have showed that beyond helping those with psychological problems, they can act as an attractor for those in need for help [39]. In addition, social media has been found to be beneficial by decreasing the distress of people with schizophrenia [50]. Our results suggest that all the eHealth resources covered may be used for providing health information to people with psychological disorders. Determining which psychological disorders respond better to each of the resources remains a future task.

Prior research has shown that patients with cardiovascular diseases constitute one group that benefits, in part through improved disease management, from telemedicine and eHealth interventions [42]. We found that patients who had cardiovascular diseases were associated with a preference for mobile apps (OR 1.12) and social media (if they had high SES; OR 3.39; see Multimedia Appendix 6). Our study complements previous findings by showing that mobile apps and social media might be the most appropriate eHealth resources for interventions for providing health information to patients with cardiovascular problems.

Concerning respiratory diseases, SMS messages, WhatsApp, and Facebook have been mentioned as useful tools for receiving health information about chronic obstructive pulmonary disease (COPD) and also for communicating with a doctor [51]. However, in our study, respiratory diseases alone were not significantly associated with a preference for any specific eHealth resource. Only for the subgroups of retired participants and participants with medium-high household income, was it possible to determine that social media or search engines, respectively, were preferred by individuals suffering from respiratory diseases. A possible explanation for the lack of significance of eHealth resources may be the lack of impact of eHealth interventions on patients with this type of diseases. In fact, the Cochrane reviews in the studies by McCabe et al and Marcano et al did not find any statistical significance in the use of mobile technology for the management of people with COPD and asthma, respectively [52,53]. Future works should focus on specific respiratory diseases to determine if these findings are applicable to all of them or whether there are differences across patients with different respiratory conditions.

Cancer was a significant predictor of the use of search engines, that is, general searches for health information on the internet. Cancer was not associated with the use of other eHealth resources. Previous studies have already shown that the use of eHealth among cancer patients is extensive [33]. Our results complement these studies by helping to understand which particular eHealth resource should be used for cancer. Currently, there is a high availability of eHealth resources for cancer. For example, there are many mobile apps concerning cancer [54]. However, the analyses of Bender et al [54] and Giunti et al [55] show that information apps about cancer are much more common than disease management ones. This is aligned with our findings. We believe that this points out that the demand for eHealth resources that provide information is higher than the demand for eHealth resources for disease management among cancer patients. Therefore, eHealth interventions should focus on providing information by, for example, pointing patients to high-quality websites about cancer.

Patients that suffered from conditions included in the group of *other diseases* (arthrosis, rheumatoid arthritis, diabetes, kidney disease, migraine, or chronic pain) were more likely to use internet videos, search engines, and mobile apps (if they were part-time workers) as eHealth resources but less likely to use social media. Some of the diseases contained in our generic group (*other diseases*) have been reported to benefit from the use of smartphone apps [30-32]. Our findings suggest that for those diseases, apps (significant only for part-time workers), in addition to videos and search engines, are appropriate resources to provide eHealth.

For patients that suffer from conditions contained in the group of *other diseases*, in some cases, our findings are not consistent with the previous literature. Prior studies have reported differences in the benefits of technology for the different diseases contained in this group. For example, mobile apps have been reported as inadequate for patients with chronic diseases [56]. For patients with chronic pain, Merolli et al found that social media was beneficial [57]; also, Hou et al found that mobile apps had a small [31] or no improvement at all on the self-management of diabetes [30]. A possible cause for the contradictions of our results with some of the previous studies is that the diseases contained in this group are very heterogeneous. Therefore, our conclusions for this group should be taken with caution. Future research should examine if differences exist across these diseases and the preference for different eHealth resources of those affected by them.

### Limitations

There are several limitations in this study that should be considered. The logistic regression model for search engines is not a robust model because the residual in the analysis of deviance is significant. This makes sense because, as stated in the Introduction section, search engines are the first input for searching information [58]. Thus, there may be very disparate factors that influence the use of search engines that are unavailable to us.

Another limitation is that, to our knowledge, it is unknown if users in the higher age groups differentiate well in their responses to the questionnaire between accessing health information from a browser on the mobile phone, an app, videos, or social media. Those with limited eHealth literacy may confuse one with another, which may lead to a high variability in the results of this part of the study. For example, search engines could be used by participants to find other resources such as social media or videos; therefore, the large use of search engines could be misleading, and some of the participants considered as search engine users may in fact have used other types of eHealth resources. In our study, we have only analyzed the use of different eHealth services (or channels), and the actual content of these services is not known.

*Psychological problems* is a crude categorization, and the group is likely to be quite heterogenous—which again may influence the outcome in terms of eHealth services used. Unfortunately, we do not have more detailed information about the types of psychological problems of the participants. However, it is reasonable to assume that most participants who had such problems had less serious psychological problems as these are the most prevalent in the general population. It is also likely that there is a participation bias in that those who suffered from the most serious psychological problems (ie, psychosis and severe depression) did not participate in the study as participation required a relatively large effort (completing a long questionnaire and attending a study center for more testing).

We lack information about the current availability of different eHealth resources for different patient groups. It is, therefore, difficult to infer from our findings whether increased use of a particular type of eHealth service may be related to a higher availability of a particular type of service.

Our results indicate which eHealth resources are more commonly used by people in each disease group. This does not necessarily mean that this eHealth resource is the best one for patients suffering from that disease. Some other factors that are relevant when planning eHealth services for different patient groups are the characteristics of the diseases and the users’ health and the users’ eHealth literacy level. Nevertheless, even in those cases where the most frequently used resource for a group of patients may not be the optimal one, our results can be used to reach those patients in the first place and redirect them to the optimal eHealth resource for a specific intervention.

As indicated in the study by Wynn et al (part 1), although the population in Tromsø may be representative of the Norwegian population, caution should be taken when extrapolating the findings to other populations [1]. This is the first time eHealth questions have been included in the Tromsø Study, and these items have, therefore, not been formally tested for validity and reliability—this will be a future task of the eHealth study group.

### Conclusions

Our findings show that different diseases influence the use of different eHealth resources. This is an important finding for health organizations to plan eHealth interventions more effectively by taking into account which type of eHealth resource should be used for each patient group. It is not clear why people with specific illnesses currently seem to favor specific eHealth resources, and it may be related to the current availability of high-quality information on different resources. However, certain eHealth resources may be better suited to specific patient groups. For instance, social media is experienced as the most useful eHealth resource for people with psychological problems. Further studies are needed to examine the underlying reasons why different patient groups prefer one type of eHealth resource over another.

## Appendix

Multimedia Appendix 1 Demographic data and diseases related to the participants that used any electronic health resource.

Multimedia Appendix 2 Logistic regression for the use of any electronic health resource.

Multimedia Appendix 3 Logistic regression for mobile apps.

Multimedia Appendix 4 Logistic regression for search engines.

Multimedia Appendix 5 Logistic regression for video services.

Multimedia Appendix 6 Logistic regression for social media.

## Abbreviations

eHealth: electronic health
kr: Norwegian Kroner
OR: odds ratio
Tromsø 7: seventh survey of the Tromsø Study
