# Supplementary material for: Impact of Illness on Electronic Health Use (The Seventh Tromsø Study - Part 2): Population-Based Questionnaire Study
Source: J Med Internet Res. 2020 Mar 5;22(3):e13116. doi: 10.2196/13116 (PMC7082738; doi:10.2196/13116)
Supplement: Multimedia Appendix 1 [file jmir_v22i3e13116_app1.docx]

Multimedia Appendix 1

Demographic data and diseases related to the participants that used any e-health resource. Missing values indicated as NA.

| **Variable** | **Category** | **Value** |
| --- | --- | --- |
| Sex: n (%)  NA = 0 | Female | 11074 (52.5%) |
|  | Male | 10009 (47.5%) |
| Age in years: mean (SD)  NA = 0 |  | 57.32 (11.3) |
| Education level: n (%)  NA = 382 | Primary or secondary | 4794 (23.16%) |
|  | Upper secondary | 5751 (27.78%) |
|  | Less than four years of college | 4007 (19.36%) |
|  | Four years or more college | 6145 (29.69%) |
| Household income per year: n (%)  NA = 897 | < 350,000 NOK  (35,000$) | 2632 (13.06%) |
|  | 350,000 – 550,000 NOK  (350,000-55,000$) | 4218 (20.9%) |
|  | 551,000 – 750,000 NOK  (55,100-75,000) | 3571 (17.7%) |
|  | 751,000 – 1,000,000 NOK  (75,100-100,000) | 4739 (23.49%) |
|  | - >1,000,000 NOK - (>100,000) | 5014 (24.85%) |
| Occupation: n (%)  NA = 331 | Full time work | 12048 (58.05%) |
|  | Part time work | 1662 (8%) |
|  | Unemployed | 137 (0.7%) |
|  | Housekeeping | 132 (0.63%) |
|  | Retired | 4787 (23.07%) |
|  | Student/Military service | 60 (0.29%) |
|  | Disability benefit | 1926 (9.3%) |
| Patient groups (by type of disease) | Have no disease | 5498 (26.077%) |
|  | Cardiovascular diseases | 7169 (34%) |
|  | Cancer | 1636 (7.76%) |
|  | Psychological problems | 2723 (12.91%) |
|  | Respiratory diseases | 2738 (12.99%) |
|  | Other diseases (rheumatoid arthritis, arthrosis, diabetes, kidney disease, migraine, chronic pain) | 11109 (52.69%) |
|  | **Total number of participants with some disease** | **15,585 (8,565 men, 7020 women) (73.92%)** |
|  | Use of mobile apps | 2687 (12.74%) |
| **Use of health resources** | Use of search engines | 10106 (47.93%) |
|  | Use of video services | 969 (4.6%) |
|  | Use of social medias | 1421 (6.74%) |
|  | **Total use some e-health resource** | **10604 (50.29%)** |
|  | Use some e-health resource and suffered from some disease | 7,854 (37.25%) |
