# Supplementary material for: Impact of Illness on Electronic Health Use (The Seventh Tromsø Study - Part 2): Population-Based Questionnaire Study
Source: J Med Internet Res. 2020 Mar 5;22(3):e13116. doi: 10.2196/13116 (PMC7082738; doi:10.2196/13116)
Supplement: Multimedia Appendix 2 [file jmir_v22i3e13116_app2.docx]

Multimedia Appendix 2

Logistic Regression for the use of any e-health resource. Missing values indicated as **‘**NA**’**. Significance at 95% is indicated as “*”.

| **Potential predictors of apps use (count)** | **Use of e-health (one time or more, any type of e-health)** | | | **Multivariable logistic regression** | |
| --- | --- | --- | --- | --- | --- |
|  | **Total** | **Ever use** | **Never** | Odds ratio (95% CI) | P-value |
| **Age*** | - | - | - | 0.94 (CI, 0.93 -0.95) | <.001 |
| **Sex*** |  |  |  |  | <.001 |
| 0 | 11074 | 6116 | 4958 | - | - |
| 1 | 10009 | 4488 | 5521 | 0.30 (CI, 0.21 - 0.44) | - |
| **Live with spouse** |  |  |  |  | <.001 |
| 0 | 4607 | 2190 | 2417 |  | - |
| 1* | 15277 | 7877 | 7400 | 0.84 (CI, 0.76 -0.92) | - |
| NA | 1199 | 537 | 662 |  | - |
| **Support from friends** |  |  |  |  | <.001 |
| 0 | 18453 | 1178 | 1032 |  | - |
| 1* | 2210 | 9285 | 9168 | 0.81 (CI, 0.73 -0.90) | - |
| NA | 420 |  | 279 |  | - |
| **Education*** |  |  |  |  |  |
| Primary/partly secondary education. (Up to 10 years of schooling) | 4794 | 1284 |  | - | - |
| Upper secondary education: (a minimum of 3 years)* | 5751 | 2699 | 3510 | 2.46 (CI 2.28 - 2.65) | <.001 |
| Tertiary education, short (less 4 years college)* | 4007 | 2353 | 1654 | 0.88 (CI 0.82 -0.94) | <.001 |
| College/university 4 years or more | 6145 | 4181 | 1964 | 1.00 (CI, 0.94 -1.07) | .99 |
| NA | 386 | 87 | 299 |  |  |
| **Occupation** * |  |  |  |  |  |
|  |  |  |  |  |  |
| full time | 12048 | 7209 | 4839 | - | - |
| part time | 1662 | 947 | 715 | 0.95 (CI, 0.77 - 1.15) | .59 |
| unemployed | 137 | 84 | 53 | 1.15 (CI, 0.63 -2.12) | .64 |
| housekeeping | 132 | 40 | 92 | 1.38 (CI, 0.63 - 2.88) | .40 |
| Retired* | 4787 | 1294 | 3493 | 1.37 (CI, 1.18 - 1.60) | <.001 |
| Student/military service | 60 | 41 | 19 | 1.26 (CI, 0.50 - 3.40) | .63 |
| Disability benefit recipient/work assessment allowance* | 1901 | 965 | 936 | 1.37 (CI 1.05 - 1.78) | .02 |
| Family income supplement | 25 | 9 | 16 | 0.87 (CI, 0.09 - 5.81) | .89 |
| NA | 331 | 15 | 316 |  |  |
| **Household income*** |  |  |  |  |  |
| 0-150,000  (0-15,000$) | 210 | 51 | 159 | **-** |  |
| 150,000-250,000*  (15,000-25,000$) | 989 | 230 | 759 | 2.01 (CI, 1.48 - 2.74) | <.001 |
| 251,000-350,000  **(**25,100-35,000$**)** | 1438 | 482 | 956 | 0.87 (CI, 0.65 -1.15) | .32 |
| 351000-450000  **(**35,100-45,000$**)** | 1906 | 835 | 1071 | 1.04 (CI, 0.82 -1.34) | .72 |
| 451000-550000  **(**45,100-55,000$**)** | 2312 | 1117 | 1195 | 0.93 (CI, 0.76 -1.15) | .53 |
| 551,000-750,000  **(**55,100-75,000$**)** | 3571 | 1752 | 1819 | 0.94 (CI, 0.79 -1.12) | .50 |
| 751,000-1,000,000  (75,100-100,000$) | 4739 | 2731 | 2008 | 1.09 (CI, 0.94 -1.27) | .26 |
| More than 1,000,000  (>100,000$) | 5014 | 3178 | 1836 | 0.95 (CI, 0.83 -1.09) | .50 |
| NA | 904 | 228 | 676 | **-** | **-** |
| **Cancer (not significant alone)** |  |  |  |  | .36 |
| 0 | 19447 | 9879 | 9568 |  | - |
| 1 | 1636 | 725 | 911 | 1.08 (CI, 0.91 - 1.29) | - |
| **Psychological problems** |  |  |  |  | <.001 |
| 0 | 18360 | 8851 | 9509 |  | - |
| 1* | 2723 | 1753 | 970 | 1.60 (CI, 1.38 - 1.84) | - |
| **Cardiovascular diseases (not significant alone)** |  |  |  |  | 0.84 |
| 0 | 13914 | 7544 | 6370 |  | - |
| 1 | 7169 | 3060 | 4109 | 0.99 (CI, 0.86 - 1.13) | - |
| **Other diseases** |  |  |  |  | <.001 |
| 0 | 9974 | 4840 | 5134 |  | - |
| 1* | 11109 | 5764 | 5345 | 1.38 (CI, 1.27 -1.50) | - |
| **Other diseases * Occupation_part_time** | - | - | - | 1.29 (CI, 1.01 - 1.64) | <.001 |
| **Sex x cancer*** | - | - | - | 1.41 (CI, 1.11 -1.80) | <.001 |
| **Cardiovascular diseases x household_income 150,000-250,000*** | - | - | - | 1.78 (CI, 1.08 - 3.01) | .03 |
| **Age x Sex*** | - | - | - | 1.01 (CI, 1.01 -1.02) | <.001 |
