# Supplementary material for: Impact of Illness on Electronic Health Use (The Seventh Tromsø Study - Part 2): Population-Based Questionnaire Study
Source: J Med Internet Res. 2020 Mar 5;22(3):e13116. doi: 10.2196/13116 (PMC7082738; doi:10.2196/13116)
Supplement: Multimedia Appendix 3 [file jmir_v22i3e13116_app3.docx]

Multimedia Appendix 3

Logistic Regression for mobile apps. Missing values indicated as NA. Significance at 95% is indicated as “*”.

| **Potential predictors of apps use (count)** | **Use of apps (one time or more)** | **Multivariable logistic regression** |
| --- | --- | --- |

|  | **Ever use** | **Never** | Odds ratio (95% CI) | P-value |
| --- | --- | --- | --- | --- |
| **Age*** | - | - | 0.97 (CI, 0.95- 0.97) | <.001 |
| **Psychological problems*** | 469 | 2254 | 1.39 (CI, 1.23-1.56) | <.001 |
| **Cardiovascular diseases*** | 882 | 6347 | 1.12 (CI, 1.01-1.24) | 0.04 |
| **Occupation** |  |  | - | - |
| full time | 1212 | 6651 | - | - |
| part time* | 210 | 1143 | 0.61 (CI, 0.40 - 0.91) | .02 |
| unemployed | 20 | 88 | 0.83(CI, 0.28 -1.97) | .70 |
| housekeeping | 10 | 99 | 0.65 (CI 0.10 - 2.26) | .57 |
| retired | 292 | 3750 | 0.97 (CI, 0.74 - 1.27) | .86 |
| Student/military service | 9 | 34 | 0.38 (CI, 0.02 - 2.03) | .36 |
| Disability benefit recipient/work assessment allowance | 224 | 1557 | 0.77 (CI, 0.51 - 1.12) | .19 |
| Family income supplement | 2 | 20 | 0.00 (NA - 0.31) | .92 |
| NA (n=264) | 3 | 261 | - | - |
| Other diseases * part time work | - | - | 2.08 (CI, 1.35 - 3.32) | .92 |
