# Supplementary material for: Impact of Illness on Electronic Health Use (The Seventh Tromsø Study - Part 2): Population-Based Questionnaire Study
Source: J Med Internet Res. 2020 Mar 5;22(3):e13116. doi: 10.2196/13116 (PMC7082738; doi:10.2196/13116)
Supplement: Multimedia Appendix 4 [file jmir_v22i3e13116_app4.docx]

Multimedia Appendix 4

Logistic Regression for search engines. Missing values indicated as NA. Significance at 95% is indicated as “*”.

| **Potential predictors of search engines use (count)** | **Use of search engines (one time or more)** | **Multivariable logistic regression** |
| --- | --- | --- |

|  | **Ever use** | **Never** | **Confidence Interval** | **P-value** |
| --- | --- | --- | --- | --- |
| **AGE_T7*** | - | - | 0.94 (CI, 0.93 - 0.95) | <.001 |
| **SEX_T7** |  |  |  | <.001 |
| 0 | 4460 | 4105 |  |  |
| 1* | 3008 | 4012 | 0.32 (CI, 0.21 - 0.50) |  |
| **LIVE_WITH_SPOUSE** |  |  |  | <.001 |
| 0 | 1638 | 1985 |  |  |
| 1* | 5455 | 5583 | 0.82 (CI, 0.73 - 0.92) |  |
| **EDUCATION** |  |  |  |  |
| Primary/partly secondary education. (Up to 10 years of schooling) | 932 | 2961 | - | - |
| Upper secondary education: (a minimum of 3 years)* | 1964 | 2332 | 2.54 (CI, 2.33 - 2.77) | < .001 |
| Tertiary education, short: College/university less than 4 years* | 1653 | 1202 | 0.85 (CI, 0.79, 0.92) | < .001 |
| College/university 4 years or more | 2871 | 1388 | 1.01 (CI, 0.94 - 1.09) | .79 |
| NA | 48 | 234 | - | - |
| **SUPPORT_FRIENDS** |  |  |  | < .001 |
| 0 | 954 | 897 |  |  |
| 1* | 6424 | 6999 | 0.80 (CI, 0.71, 0.90) |  |
| **PSYCHO_DIS_BIN1** |  |  |  | < .001 |
| 0 | 5794 | 7968 |  |  |
| 1* | 1674 | 1049 | 1.39 (CI, 1.25 - 1.55) |  |
| **CANCER_DIS_BIN1** |  |  |  | < .001 |
| 0 | 6790 | 7159 |  |  |
| 1* | 678 | 958 | 1.26 (CI, 1.11 - 1.44) |  |
| **OTHER_DIS_BIN1** |  |  |  | <.001 |
| 0 | 2001 | 2475 |  |  |
| 1* | 5167 | 5642 | 1.27 (CI, 1.13 - 1.42) |  |

| **CARDIOVASCULAR_DISEASE_BIN** |  |  |  | .32 |
| --- | --- | --- | --- | --- |

| 0 |  |  | - | - |
| --- | --- | --- | --- | --- |
| 1 |  |  | 0.94 (CI, 0.84 - 1.06) |  |
| RESPIRATORY_DISEASE_BIN |  |  |  | .50 |
| 0 |  |  | - | - |
| 1 |  |  | 1.06 (CI, 0.89 - 1.25) | 0.01 |
| **HOUSEHOLD INCOME** |  |  |  |  |
| 0-150,000  (0-15,000) | 39 | 134 |  |  |
| 150,000-250,000*  (15,000-25,000$) | 179 | 697 | 2.57 (1.86 - 3.60) | <.001 |
| 251,000-350,000  (25,100-35,000$) | 401 | 846 | 0.77 (0.57 - 1.04) | .01 |
| 351,000-450,000  (35,100-45,000$) | 646 | 880 | 1.06 (0.82 - 1.37) | .62 |
| 451,000-550,000  (45,100-55,000$) | 814 | 971 | 1.16 (0.94 - 1.43) | .17 |
| 551,000-750,000*  (55,100-75,000$) | 1283 | 1448 | 0.74 (0.62 - 0.87) | .00 |
| 751,000-1,000,000  (75,100-100,000$) | 1914 | 1433 | 1.10 (0.95 - 1.27) | .43 |
| More than 1,000,000  (>100,000$) | 2048 | 1148 | 1.07 (0.94 - 1.22) | .69 |
| NA | 144 | 560 |  |  |
| **OCCUPATION** |  |  |  |  |
| full time | 4724 | 3139 | - | - |
| part time | 762 | 591 | 0.99 (CI, 0.75- 1.35) | .93 |
| unemployed | 68 | 40 | 1.06 (CI, 0.51- 2.25) | .87 |
| housekeeping | 26 | 83 | 0.86(CI, 0.29 - 2.28) | .77 |
| Retired* | 980 | 3062 | 1.31 (CI, 1.07 -1.59) | .01 |
| Student/military service | 30 | 13 | 0.91 (CI, 0.24 - 4.35) | .89 |
| Disability benefit recipient/work assessment allowance | 858 | 923 | 1.30 (CI, 0.96- 1.75) | .09 |
| Family income supplement | 9 | 13 | 3.85 (CI, 0.53-34.15) | .18 |
| NA | 11 | 253 |  |  |
| **SEX:CARDIOVASCULAR*** | - | - | 1.25 (CI, 1.07 - 1.47) | .005 |
| **AGE_T7:SEX *** | - | - | 1.01 (CI, 1.01 - 1.02) | .005 |
| **OTHER_DISEASE:OCCUPATION** | - | - |  |  |
| full time | - | - | - | - |
| part time | - | - | 1.21 (CI, 0.88 - 1.65) | .24 |
| unemployed | - | - | 1.73 (CI, 0.68 -4.40) | .25 |
| housekeeping | - | - | 0.73 (CI, 0.23-2.496) | .60 |
| Retired* | - | - | 0.69 (CI, 0.56- 0.84) | <.001 |
| Student/military service | - | - | 0.98 (CI, 0.17-4.81) | .98 |
| Disability benefit recipient/work assessment allowance | - | - | 0.83 (CI, 0.60-1.15) | .27 |
| Family income supplement | - | - | 0.12 (CI, 0.01-1.40) | .097 |
| **RESPIRATORY: HOUSEHOLD_INCOME** |  |  |  |  |
| 150,000-250,000  (0-18000$) | - | - | 0.87 (CI, 0.47-1.65) | .67 |
| 251,000-350,000  (18120-30000$) | - | - | 1.03 (CI, 0.55 - 1.90) | .93 |
| 351,000-450,000  (30120-42000$) | - | - | 0.94 (CI, 0.55-1.62) | .82 |
| 451,000-550,000  (30120-54000$) | - | - | 0.88 (CI, 0.57-1.37) | .58 |
| 551,000-750,000*  (66120-90000$) | - | - | 1.65 (CI, 1.14-2.38) | .01 |
| 751,000-1,000,000  (90120-120000$) | - | - | 0.82 (CI, 0.60-1.14) | .24 |
| More than 1,000,000*  (>120000$) | - | - | 0.67 (CI, 0.49-0.92) | .01 |
