# Supplementary material for: Impact of Illness on Electronic Health Use (The Seventh Tromsø Study - Part 2): Population-Based Questionnaire Study
Source: J Med Internet Res. 2020 Mar 5;22(3):e13116. doi: 10.2196/13116 (PMC7082738; doi:10.2196/13116)
Supplement: Multimedia Appendix 5 [file jmir_v22i3e13116_app5.docx]

Multimedia Appendix 5

Logistic Regression for video services. Missing values indicated as NA. Significance at 95% is indicated as “*”.

| **Potential predictors of Internet video use (count)** | **Use of Internet videos (one time or more)** | **Multivariable logistic regression** |
| --- | --- | --- |

|  | **Ever use** | **Never** | **Confidence Interval** | **P-value** |
| --- | --- | --- | --- | --- |
| **AGE_T7** | - | - | 0.97 (CI, 0.96-0.98) | <0.001 |

| **EDUCATION** |  |  |  |  |
| --- | --- | --- | --- | --- |

| Primary/partly secondary education. (Up to 10 years of schooling) | 93 | 3800 | - | - |
| --- | --- | --- | --- | --- |
| Upper secondary education: (a minimum of 3 years)* | 192 | 4104 | 2.16 (CI, 1.79-2.61) | .01 |
| Tertiary education, short: College/university less than 4 years* | 183 | 2672 | 0.81 (CI, 0.68-0.95) | .01 |
| Tertiary education, long: College/university 4 years or more | 295 | 3964 | 0.99 (CI, 0.85-1.15) | .90 |
| NA | 4 | 278 |  |  |
| **SEX_T7** |  |  |  | <.001 |
| 0 | 407 | 8158 |  |  |
| 1* | 360 | 6660 | 1.31 (CI, 1.13-1.53) |  |
| **PSYCHOLOGICAL PROBLEMS** |  |  |  | <.001 |
| 0 | 544 | 12318 |  |  |
| 1* | 223 | 2500 | 1.70 (CI, 1.43 -2.01) |  |
| **OTHER DISEASE** |  |  |  | <.001 |
| 0 | 181 | 4295 |  |  |
| 1* | 586 | 10523 | 1.43 (CI, 1.19-1.71) |  |
| **HOUSEHOLD_INCOME_T7_REF** |  |  |  | <.001 |
| 0-150,000  (0-15,000) | 13 | 160 |  |  |
| 150,000-250,000*  (15,000-25,000$) | 35 | 841 | 0.45 (CI, 0.31-0.67) | <.001 |
| 251,000-350,000  (25,100-35,000$) | 49 | 1198 | 1.19 (CI, 0.81-1.68) | .35 |
| 351,000-450,000  (35,000-45,000$) | 70 | 1456 | 0.76 (CI, 0.55-1.07) | .10 |
| 451,000-550,000  (45,100-55,000$) | 105 | 1680 | 1.09 (CI, 0.81-1.45) | .57 |
| 551,000-750,000  (55,100-75,000$) | 123 | 2608 | 1.005 (CI, 0.77-1.30) | .97 |
| 751,000-1,000,000  (75,100-100,000$) | 187 | 3160 | 0.89 (CI, 0.67-1.14) | .36 |
| More than 1,000,000  (>100,000$) | 169 | 3027 | 0.87 (CI, 0.70-1.10) | .25 |
| NA | 16 | 688 |  |  |
