# Supplementary material for: Impact of Illness on Electronic Health Use (The Seventh Tromsø Study - Part 2): Population-Based Questionnaire Study
Source: J Med Internet Res. 2020 Mar 5;22(3):e13116. doi: 10.2196/13116 (PMC7082738; doi:10.2196/13116)
Supplement: Multimedia Appendix 6 [file jmir_v22i3e13116_app6.docx]

Multimedia Appendix 6

Logistic Regression for social media. Missing values indicated as NA. Significance at 95% is indicated as “*”.

| **Potential predictors of social media use (count)** | **Use of social media (one time or more)** | **Multivariable logistic regression** |
| --- | --- | --- |

|  | **Ever use** | **Never** | **Confidence Interval** | **P-value** |
| --- | --- | --- | --- | --- |
| **AGE_T7*** | - | - | 0.96 (CI, 0.95-0.97) | <.001 |
| **SEX_T7** |  |  | - | <.001 |
| 0 | 816 | 7749 | - |  |
| 1* | 329 | 6691 | 0.54 (CI, 0.43-0.62) |  |
| **Psychological problems** |  |  |  | <.001 |
| 0 | 809 | 12053 | - |  |
| 1* | 336 | 2387 | 1.65 (CI, 1.42-1.91) |  |
| **Household income** |  |  |  |  |
| 0-150,000  (0-15,000$) | 16 | 157 | - |  |
| 151,000-250,000*  (15,100-25,000$) | 54 | 822 | 0.43 (CI, 0.27- 0.71) | <.001 |
| 251,000-350,000  (25,100-35,000$) | 97 | 1150 | 1.17 (CI, 0.73-1.79) | .49 |
| 351,000-450,000  (35,100-45,000$) | 135 | 1391 | 0.72 (CI, 0.49-0.11) | .098 |
| 451,000-550,000  (45,100-55,000$) | 161 | 1624 | 1.01 (CI, 0.72-1.42) | .95 |
| 551,000-750,000  (55,100-75,000$) | 194 | 2537 | 0.91 (CI, 0.67-1.23) | .56 |
| 751,000-1,000,000  (75,100-100,000$) | 248 | 3099 | 0.95 (CI, 0.72-1.25) | .73 |
| More than 1,000,000  (>100,000$) | 201 | 2995 | 1.06 (CI, 0.82-1.37) | .67 |
| NAs | 39 | 665 |  |  |
| **Occupation** |  |  | - |  |
| Full time | 579 | 7284 | - | - |
| Part time* | 150 | 1203 | 1.42(CI, 1.13-1.76) | .001 |
| Unemployed | 12 | 96 | 1.35 (CI, 0.62-2.63) | .41 |
| Housekeeping | 9 | 100 | 1.43 (CI, 0.58-3.03) | .39 |
| Retired | 186 | 3856 | 1.06 (CI, 0.79-1.41) | .68 |
| Student/military service | 6 | 37 | 1.06 (CI, 0.36-2.54) | .896 |
| Disability benefit recipient/work assessment allowance* | 198 | 1583 | 1.42(CI, 1.14-1.76) | .002 |
| Family income supplement | 2 | 20 | 1.50 (CI, 0.08-9.06) | .71 |
| NAs | 3 | 261 | - | - |
| **EDUCATION** |  |  | **-** |  |
| Primary/partly secondary education. (Up to 10 years of schooling) | 214 | 3679 | **-** | **-** |
| Upper secondary education: (a minimum of 3 years)* | 354 | 3942 | 1.19 (CI, 1.03-1.38) | .02 |
| Tertiary education, short: College/university less than 4 years* | 231 | 2624 | 0.81 (CI, 0.71-0.92) | .002 |
| Tertiary education, long: College/university 4 years or more | 339 | 3920 | 1.06 (CI, 0.94-1.21) | .33 |
| NAs | 7 | 275 | **-** | **-** |
| Sex x Household income 751,000-1000000* | - | - | 1.67 (CI, 1.07-2.60) | .02 |
| Household income 151,000-250,000 x cardiovascular disease* | - | - | 3.39 (1.62-8.16) | .002 |
| Respiratory disease x retired* | - | - | 1.96 (1.28 - 2.97) | .001 |
